# Editorial: The Role of Microenvironment in the Homing, Maintenance, and Release of Glioma Stem-Like Cells

**DOI:** 10.3389/fonc.2018.00007

**Published:** 2018-01-30

**Authors:** Shwetal Mehta

**Affiliations:** ^1^Barrow Brain Tumor Research Center, Barrow Neurological Institute (BNI), Phoenix, AZ, United States

**Keywords:** glioma stem cells, microenvironment, glioblastoma, stemness, neurooncology

**Editorial on the Research Topic**

**The Role of Microenvironment in the Homing, Maintenance, and Release of Glioma Stem-Like Cells**

Among brain tumors, gliomas are the most common primary tumors in adults (1). Glioblastomas (GBMs) are the most aggressive and lethal gliomas with a median survival of 12–15 months (2). One of the challenges in the treatment of GBMs is the presence of a subpopulation of treatment-resistant cells with stem-like properties referred to as glioma stem cells (GSCs) (3–5). GSCs have the capacity to self-renew, to differentiate into various central nervous system cell types in presence of appropriate environmental cues and are highly infiltrative. GSCs have increased DNA repair capacity and express multidrug resistance pumps that impart resistance to therapies (6). Data from several studies suggest that GSCs are maintained in specialized niches that help maintain GSCs in a replication competent state or in a quiescent state enabling them to evade cytotoxic therapies (7, 8). In this research topic, we focus on mechanisms that help the homing of GSCs within glioma niches, maintenance of their self-renewal capacity and release of GSCs into the tumor microenvironment.

Two major components of the microenvironment that affect GSC biology are the vasculature and the cells of the immune system. In this research topic, Audia et al. provide an extensive review of current literature on the cross-regulatory interactions between the various vascular cell types and GSCs as well as the immune system and GSCs. Roos et al. further provide insight into the roles of astrocytes and extracellular matrix factors in promoting GSC invasion and migration through the JAK/STAT pathway. In addition, this review also provides insight into how hypoxia and tissue pH affects GSC maintenance through upregulation of HIF2α and stemness genes. One of the areas of active research is to identify the mechanisms through which GSCs interact with the tumor microenvironment. Mondal et al. have reviewed recent studies on the extravesicular transport of molecules between the microenvironment and GSCs that affect various aspects of GSC biology including survival, invasion, and therapy resistance. Extracellular vesicles (EVs) have been shown to transport a wide variety of molecules including signaling proteins, lipids, mRNA, miRNA, and DNA. The minireview by Mondal et al. elaborates on these diverse roles of EVs in promoting glioma invasion, metabolic regulation of hypoxia signaling, and modulating the immune response.

The most recent genomic classification of GBM based on single cell gene expression has defined three subtypes based on their transcriptional profiles designated as proneural, classical, and mesenchymal (9). In this study, a robust analysis of single cells from three patients was able to rule out neuronal subtype as contamination from normal brain and confirms that the mesenchymal subtype of GBM has increased infiltration of tumor-associated macrophages and microglia. In an original research article in this research topic, Sharma et al. attempted to identify a tumor-intrinsic immunohistochemistry-based minimal multigene signature to differentiate between proneural and mesenchymal subtypes of GBM. Using two patient-derived samples they identified four genes linked to angiogenesis that are highly elevated in mesenchymal GBMs compared with proneural subtypes.

In summary, this research topic highlights the significance of the various microenvironmental factors in the regulation of GSC biology. Future research focused on the interactions between the cancer cells and the surrounding niche will not only advance our understanding of this deadly disease but will also provide insights into patients who might benefit from immune modulation.

## Author Contributions

The author confirms being the sole contributor of this work and approved it for publication.

## Conflict of Interest Statement

The author declares that the research was conducted in the absence of any commercial or financial relationships that could be construed as a potential conflict of interest. The handling editor declared a shared affiliation, though no other collaboration, with the author.
